# A Deep Learning Image Reconstruction Algorithm for Improving Image Quality and Hepatic Lesion Detectability in Abdominal Dual-Energy Computed Tomography: Preliminary Results

**DOI:** 10.1007/s10278-023-00893-y

**Published:** 2023-08-14

**Authors:** Bingqian Chu, Lu Gan, Yi Shen, Jian Song, Ling Liu, Jianying Li, Bin Liu

**Affiliations:** 1 Present Address: Department of Radiology, the First Affiliated Hospital of Anhui Medical University, Heifei 230022, People’s Republic of China; 2 Department of Radiology, Huainan Oriental Guangji Hospital, Huainan 232101, People’s Republic of China; 3 CT Research Center, GE Healthcare China, Shanghai 210000, People’s Republic of China

**Keywords:** Adaptive statistical iterative reconstruction, Computed tomography, Deep learning, Dual-energy CT, Image reconstruction

## Abstract

This study aimed to compare the performance of deep learning image reconstruction (DLIR) and adaptive statistical iterative reconstruction-Veo (ASIR-V) in improving image quality and diagnostic performance using virtual monochromatic spectral images in abdominal dual-energy computed tomography (DECT). Sixty-two patients [mean age ± standard deviation (SD): 56 years ± 13; 30 men] who underwent abdominal DECT were prospectively included in this study. The 70-keV DECT images in the portal phase were reconstructed at 5-mm and 1.25-mm slice thicknesses with 40% ASIR-V (ASIR-V40%) and at 1.25-mm slice with deep learning image reconstruction at medium (DLIR-M) and high (DLIR-H) levels and then compared. Computed tomography (CT) attenuation, SD values, signal-to-noise ratio (SNR), and contrast-to-noise ratio (CNR) were measured in the liver, spleen, erector spinae, and intramuscular fat. The lesions in each reconstruction group at 1.25-mm slice thickness were counted. The image quality and diagnostic confidence were subjectively evaluated by two radiologists using a 5-point scale. For the 1.25-mm images, DLIR-M and DLIR-H had lower SD, higher SNR and CNR, and better subjective image quality compared with ASIR-V40%; DLIR-H performed the best (all *P* values < 0.001). Furthermore, the 1.25-mm DLIR-H images had similar SD, SNR, and CNR values as the 5-mm ASIR-V40% images (all *P* > 0.05). Three image groups had similar lesion detection rates, but DLIR groups exhibited higher confidence in diagnosing lesions. Compared with ASIR-V40% at 70 keV, 70-keV DECT with DLIR-H further reduced image noise and improved image quality. Additionally, it improved diagnostic confidence while ensuring a consistent lesion detection rate of liver lesions.

## Introduction

Hepatic lesions often represent as space-occupying lesions. These lesions differ from the normal liver morphology and have a space-occupying effect according to the abnormal echo area or density area found in the liver parenchyma during liver imaging examination. They can be categorized into benign lesions (hepatic hemangiomas, cysts, etc.) and malignant lesions (hepatocellular carcinoma, etc.) [1]. As liver lesions in the early stage generally have no obvious clinical symptoms, medical imaging is an essential technology for diagnosis in clinical practice. Upper abdominal enhanced computed tomography (CT) is the most performed procedure for differentiating benign and malignant liver lesions with high sensitivity, accuracy, and specificity [2, 3].

Dual-energy CT (DECT) is an advanced technology in the field of CT wherein low- and high-energy datasets are used to generate extra quantitative parameters [iodine concentration images, virtual monochromatic images (VMIs), effective atomic numbers, etc.] and overcome the limitations of conventional CT [4, 5]. Compared with conventional CT, the VMIs at 70 keV generate the lowest noise and the highest contrast-to-noise ratio (CNR), improving the CNR of abdominal organs by 13.8–24.7%, and more accurately reflecting the true level of lesion enhancement [6, 7]. Moreover, optimal monochromatic imaging combined with iterative reconstruction can eliminate beam-hardening artifacts and improve image quality [8, 9].

Artificial intelligence-based reconstruction algorithms have recently been developed, such as deep learning image reconstruction (DLIR; TrueFidelity, GE HealthCare Waukesha, WI, USA). DLIR uses high dose and high quality FBP images as ground truth and significantly suppresses image noises and streak artifacts on various phantom studies and clinical applications [10–13]. Many studies demonstrate that DLIR notably improves image quality while maintaining or even improving diagnostic accuracy in conventional CT scanning using a single tube voltage [14–16]. The DLIR algorithm has recently been extended to DECT imaging mode [17]. However, studies on the value of DECT with DLIR in diagnosing liver lesions are relatively few. Upper abdominal CT in the portal venous phase is an essential clinical imaging technique for evaluating the portal vein, preoperative evaluation of liver transplantation, and assessment of blood supply for liver tumors [18]. Hence, this study aimed to compare the DLIR and adaptive statistical iterative reconstruction-Veo (ASIR-V) in improving image quality and diagnostic performance in the portal venous phase of abdominal DECT imaging.

## Materials and Methods

The study protocol was approved by our institutional review board (Approval Number: PJ2023-06–26), and informed written consent was obtained from all study participants.

### Study Design

This prospective study focused on the abdominal DECT images in the portal phase using the VMIs at 70 keV. The 70-keV 5-mm slice thickness images reconstructed using ASIR-V at 40% level (ASIR-V40%) were used as the reference standard. The other three image datasets were reconstructed using ASIR-V40%, DLIR at a medium level (DLIR-M), and DLIR at a high level (DLIR-H) with a slice thickness of 1.25 mm. The image noise, signal-to-noise ratio (SNR), CNR, subjective image quality, and diagnostic performance, including lesion detection rate and diagnostic confidence, of upper abdominal CT images in the portal phase were compared.

### Study Population

**Fig. 1:**
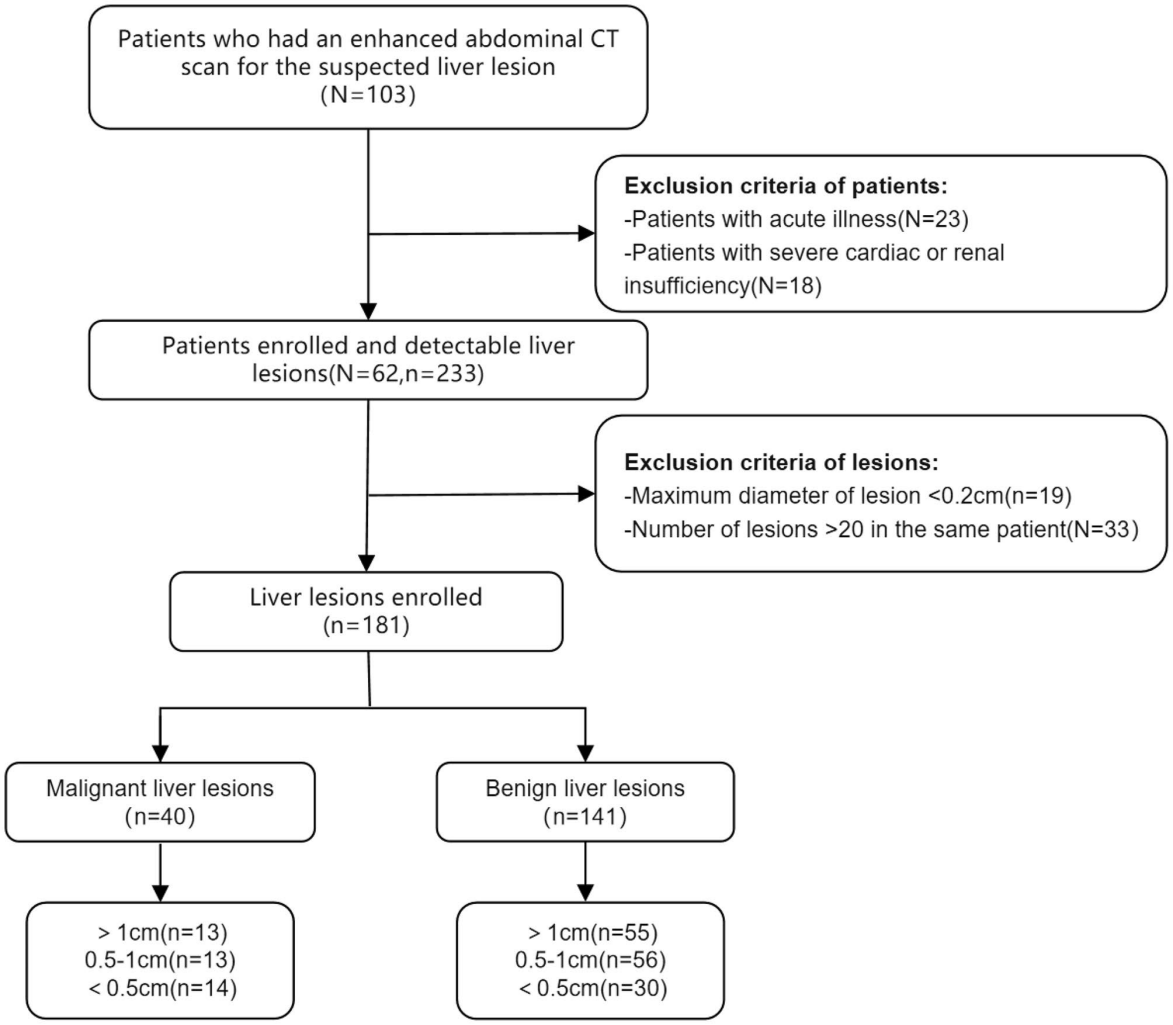
Flowchart of study population and lesion enrollment *N*, Number of patients; *n*, number of lesions

Patients suspected of having abdominal lesions indicated by the abdominal plane and enhanced CT scans at the First Affiliated Hospital of Anhui Medical University from February to April 2022 were included in this study. The inclusion criteria were as follows: (1) age ≥ 18 years and (2) men and nonpregnant women. The exclusion criteria were as follows: (1) patients with acute illness and (2) patients with severe cardiac or renal insufficiency. Finally, 62 patients (30 male and 32 female) were included in this study, with an average age of 87 ± 13.35 years (range, 29–80 years) and an average body mass index of 23.12 ± 1.75 kg/m^2^ (range, 19.4–25.5 kg/m^2^). Figure 1 shows the study flowchart.

### CT Scanning and Image Reconstruction

All data acquisitions were performed using a 256-slice CT scanner (Revolution CT, GE HealthCare, WI, USA). The scan protocols for the abdominal CT were as follows. Patients were scanned from the top of the diaphragm to the level of the iliac spine in the supine position with a DECT mode using the following parameters: fast tube voltage switching between 80 and 140 kVp during gantry rotation; noise index (NI), 9; automatic tube current selection; gantry rotation time, 0.8 s; and helical pitch, 0.992:1. Using a double-cylinder high-pressure syringe, the nonionic contrast agent Iohexol (Omnipaque 320; GE Healthcare) was injected into the right anterior elbow vein via an 18–20 G intravenous indwelling needle at a rate of 2–3 mL/s. The total amount was 0.8–1 mL/kg. The bolus tracking technique was used to determine the delay time.

Four sets of axial monochromatic images at 70 keV were generated in the portal phase of each patient: 5 mm ASIR-V40%, 1.25 mm ASIR-V40%, 1.25 mm DLIR-M, and 1.25 mm DLIR-H. These images of four groups were analyzed and compared.

### Analysis of Image Quality

#### Objective Analysis

All CT images were transferred to a PACS workstation. A radiologist who was not involved in grading the examinations measured CT value and image noise by placing a region of interest (ROI) of 20–40 mm^2^ in four standard locations: liver parenchyma, spleen, erector spinae, and intramuscular fat. The size, shape, and position of ROI were consistent among different reconstructions while avoiding artifacts and lesion areas. The data were measured in three consecutive image slices and averaged. The SNR and CNR of liver parenchyma, spleen, and erector spinal muscle were calculated using the SD of intramuscular fat as the background image noise. The corresponding formulas were as follows: $CNR=\frac{{(ROI}_{i}-{ROI}_{b})}{{SD}_{b}}, SNR=\frac{{ROI}_{i}}{{SD}_{i}}$, where ${ROI}_{i}$ denotes the CT attenuation of the interested organ, including liver parenchyma, spleen, and erector spinae; ${ROI}_{b}$ denotes the CT attenuation of intramuscular fat; ${\mathrm{SD}}_{\mathrm{b}}$ denotes the image noise of intramuscular fat; and ${\mathrm{SD}}_{\mathrm{i}}$ denotes the image noise of interested organs. The same radiologist performed all data measurements and calculations.

#### Subjective Analysis

After receiving a specific and standardized scoring criterion (Table 1), two radiologists with 23 and 10 years of experience in abdominal imaging and diagnosis analyzed images independently. No time limitation was imposed for reviewing images. Two radiologists rated the overall image quality of the four image groups in the portal phase using a 5-point Likert scale, as shown in Table 1.

**Table 1:** Grading scale for image quality

**Parameters**	**Score**				
	**1**	**2**	**3**	**4**	**5**
Vessel edge	Unacceptable	Suboptimal	Acceptable	Good	Excellent
Image noise	Unacceptable	Above average	Average	Less than average	Minimum
Overall image quality	Unacceptable	Suboptimal	Average	Above average	Superior
Influence diagnosis	Poor	Fair	Moderate	Substantial	Almost perfect

### Diagnostic Performance

#### Lesion Detection

The hepatic lesion detectability was compared in the three groups of 1.25-mm slice thickness in the portal phase (ASIR-V40%, DLIR-M, and DLIR-H at 70 keV). Two radiologists blindly performed lesion detection for the three groups of images, including locating and qualitatively analyzing all liver lesions. Benign lesions involved hepatic hemangiomas, and cysts and malignant lesions involved hepatocellular carcinoma and liver metastasis. Only lesions whose diameter ranged from 0.2 to 2 cm were labeled. The reviews among three groups of images from the same participant were performed with a time interval of 10–20 days to minimize the impact of memory. The reviews were conducted randomly.

Combining all available images and medical records of the patients (contrast-enhanced CT images in all phases and other modalities, i.e., magnetic resonance imaging and positron emission tomography/CT), a radiologist with 25 years of experience in abdominal imaging diagnosed 40 malignant liver lesions, including pathologically proven liver metastases, and 141 benign liver lesions, including hepatic cyst and hepatic hemangioma, as the reference standard. Then, all 181 lesions were classified based on the diameter: > 1, 0.5–1, and < 0.5 cm, as shown in Fig. 1.

#### Diagnostic Confidence

The diagnostic confidence was scored using a 5-point Likert scale: 1, very poor diagnostic confidence with no certainty; 2, poor diagnostic confidence; 3, moderate diagnostic confidence; 4, good diagnostic confidence; and 5, excellent diagnostic confidence with full certainty. The cases were reviewed in a random order. No time limit was imposed for reviewing, but the radiologists were required to read images in the same way as in clinical practice. The result regarding these CT characteristics was based on the agreement between the two radiologists.

### Statistical Analysis

Commercial statistical software (SPSS version 260, IBM) was used to analyze all data. The continuous data were expressed as means ± SD, and the nonparametric subjective evaluation data were reported as frequency (percentage). The Kolmogorov–Smirnov test was performed to verify the normality of continuous variables. The quantitative data with normal distribution were compared using the one-way analysis of variance, whereas non-normally distributed quantitative data were compared using the Kruskal–Wallis test. Histograms were used to illustrate the distributions of Likert scale reader scores across reconstructions. The weighted Cohen kappa statistic was used to assess agreement between the two readers. A *P* value < 0.05 indicated a statistically significant difference.

## Results

### Analysis of Image Quality

#### Objective Analysis

The objective assessments of images are presented in Table 2. According to the measured SD and calculated SNR and CNR, the order of image quality from the highest to the lowest was DLIR-H, DLIR-M, and ASIR-V40% under the same 1.25-mm thickness. The SD values for hepatic parenchyma, spleen, erector spinae, and intramuscular fat on the 70-keV 1.25-mm DLIR-H images were significantly lower than those on the 70-keV 1.25-mm ASIR-V40% and DLIR-M images (all *P* values < 0.01). The SNR and CNR values for the hepatic parenchyma, spleen, and erector spinae on the 70-keV 1.25-mm DLIR-H images were significantly higher than those on the 70-keV 1.25-mm ASIR-V40% and DLIR-M images (all *P* values < 0.01). The values of SD, SNR, and CNR on 1.25-mm DLIR-H images were similar to those on 5-mm ASIR-V40% images, except for the SD of erector spinae with a *P* value of 0.001 (Tables 3 and 4).

**Table 2:** Comparison of standard dose, SNR, and CNR of four sets of images

					**P**			
	**70 keV 5 mm** **AV40**	**70 keV 1.25 mm** **AV40**	**70 keV 1.25mmDLIR-M**	**70 keV 1.25mmDLIR-H**	**70keV1.25 mm DLIR-M Vs.70keV5mm** **AV40**	**70keV1.25 mm DLIR-H Vs.70keV5mm** **AV40**	**70keV1.25 mm DLIR-M Vs.70keV1.25 mm** **AV40**	**70keV1.25 mm DLIR-H** **Vs.70keV1.25 mm** **AV40**
Standard dose								
Liver	9.86 ± 4.08	18.48 ± 2.17	13.49 ± 2.33	9.74 ± 1.21	< 0.001	0.355	< 0.001	< 0.001
Spleen	9.58 ± 1.26	18.65 ± 4.14	13.38 ± 2.38	10.30 ± 4.18	< 0.001	0.207	< 0.001	< 0.001
Erector spinae	9.52 ± 1.43	18.95 ± 2.89	14.09 ± 2.92	10.42 ± 2.21	< 0.001	0.001	< 0.001	< 0.001
Intramuscular fat	12.06 ± 2.36	19.52 ± 3.56	15.69 ± 3.60	13.11 ± 3.66	< 0.001	0.052	< 0.001	< 0.001
SNR								
Liver	8.73 ± 2.09	5.36 ± 1.37	6.69 ± 1.89	8.22 ± 2.67	< 0.001	0.128	< 0.001	< 0.001
Spleen	9.78 ± 2.80	6.14 ± 1.44	7.72 ± 2.20	9.44 ± 3.06	< 0.001	0.418	< 0.001	< 0.001
Erector spinae	5.08 ± 1.12	3.08 ± 0.68	3.91 ± 0.98	4.73 ± 1.31	< 0.001	0.080	< 0.001	< 0.001
CNR								
Liver	16.26 ± 3.79	9.89 ± 2.12	12.52 ± 3.10	15.35 ± 4.47	< 0.001	0.213	< 0.001	< 0.001
Spleen	17.30 ± 4.55	10.66 ± 2.15	13.55 ± 3.47	16.57 ± 4.87	< 0.001	0.358	< 0.001	< 0.001
Erector spinae	12.61 ± 3.07	7.60 ± 1.52	9.74 ± 2.39	11.86 ± 3.27	< 0.001	0.208	< 0.001	< 0.001

The data are expressed as mean ± SD. Statistical significance was set at *P* < 0.05. *AV40* Adaptive statistical iterative reconstruction with Veo at the level of 40%, *DLIR-H* deep learning image reconstruction at high levels, *DLIR-M* deep learning image reconstruction at medium levels, *CNR* contrast-to-noise ratio, *SNR* signal-to-noise ratio

**Table 3:** Lesion detection rate

			**70 keV AV40**	**70 keV DLIR-M**	**70 keV DLIR-H**
Reference standard		> 1 cm	55	55	55
	Benign	0.5–1 cm	56	56	56
		< 0.5 cm	30	30	30
		> 1 cm	13	13	13
	Malignant	0.5–1 cm	13	13	13
		< 0.5 cm	14	14	14
Detected lesions		> 1 cm	55	55	55
	Benign	0.5–1 cm	56	56	56
		< 0.5 cm	28	28	28
		> 1 cm	13	13	13
	Malignant	0.5–1 cm	13	13	13
		< 0.5 cm	13	13	13
Lesion detection rate (%)		> 1 cm	100	100	100
	Benign	0.5–1 cm	100	100	100
		< 0.5 cm	93.33	93.33	93.33
		> 1 cm	100	100	100
	Malignant	0.5–1 cm	100	100	100
		< 0.5 cm	92.86	92.86	92.86

*AV40* Adaptive statistical iterative reconstruction-Veo 40%, *DLIR-H* deep learning image reconstruction at high levels, *DLIR-M* deep learning image reconstruction at medium levels

**Table 4:** Diagnostic confidence of the lesions by two radiologists

	**70 keV AV40**	**70 keV DLIR-M**	**70 keV DLIR-H**	**χ**^**2**^	**P**
Radiologist 1	3.95 ± 0.41	4.57 ± 0.50	4.64 ± 0.49	91.027	< 0.001
Radiologist 2	3.94 ± 0.38	4.43 ± 0.50	4.53 ± 0.50	80.400	< 0.001

The data are expressed as mean ± SD. Statistical significance was set at *P* < 0.05. *AV40* Adaptive statistical iterative reconstruction-Veo 40%, *DLIR-H *deep learning image reconstruction at high levels, *DLIR-M *deep learning image reconstruction at medium levels. The table shows that both readers scored the highest in the 70-keV 1.25-mm DLIR-H group regarding diagnostic confidence in the lesion

#### Subjective Analysis

**Fig. 2:**
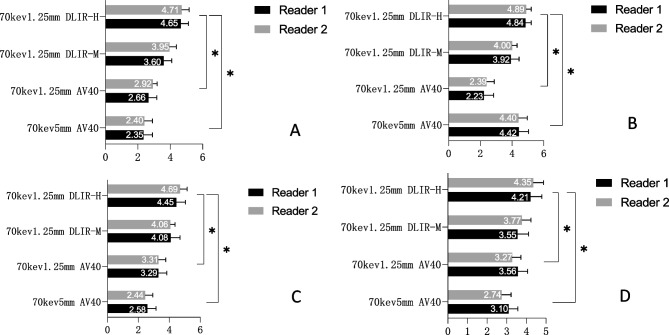
Subjective analysis of image quality. (**A**) Vessel edge; (**B**) image noise; (**C**) overall image quality; (**D**) influence of diagnosis. AV40, Adaptive statistical iterative reconstruction with Veo at a level of 40%; DLIR-H, deep learning image reconstruction at high levels; DLIR-M, deep learning image reconstruction at medium levels. **A**–**D** shows Four scores of image quality by the two physicians according to Table 1. The results showed that the 70-keV 1.25-mm DLIR-H group had higher scores than the other three groups. That is, the 70-keV 1.25-mm DLIR-H image showed a sharper vessel edge, a lower image noise, a better overall image quality, and a minimal impact on diagnosis. ^*^P < 0.05

**Fig. 3:**
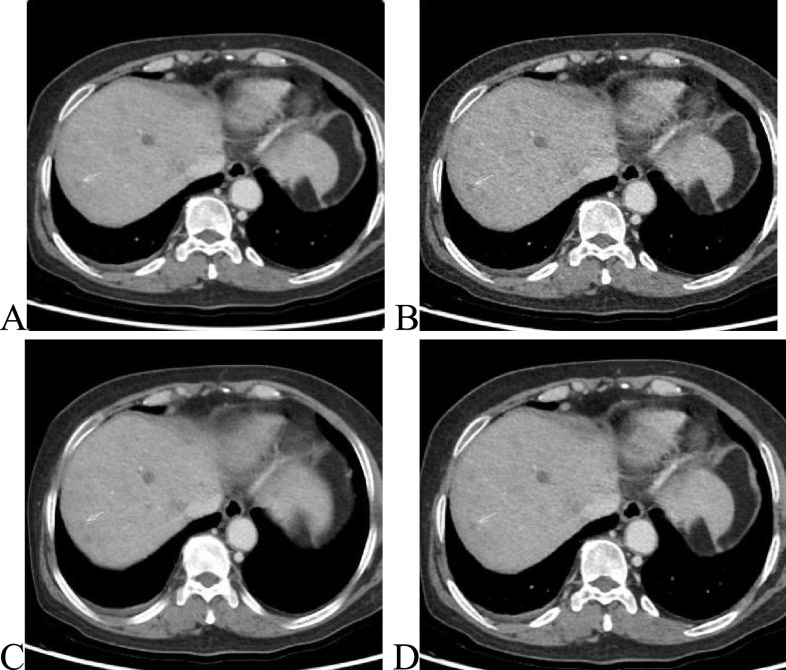
Images of a 51-year-old patient with “intrahepatic metastasis” having a body mass index of 25.4 kg/m^2^. (**A**) Image obtained from 70-keV 5-mm ASIR-V40% reconstruction. (**B**–**D**) Images obtained from 70-keV 1.25-mm ASIR-V40%, 70-keV 1.25-mm DLIR-M, and 70-keV 1.25-mm DLIR-H reconstructions in that order. The *white arrow* shows a small lesion of 0.32-cm diameter lesion in the right lobe of the liver, which, combined with multiphase enhancement, was characterized as a cyst. (**A**) A blurred border and poor contrast with the surrounding liver parenchyma, which both physicians missed it in this group. (**B**) A clearer border of the small lesion, but it had a more pronounced grainy feel. (**C** and **D**) A more pronounced contrast with the surrounding tissue, a softer image, a lower noise value, and good spatial resolution. Both physicians agreed that **D** (70-keV 1.25-mm DLIR-H) had the best image quality and the highest diagnostic confidence

**Fig. 4:**
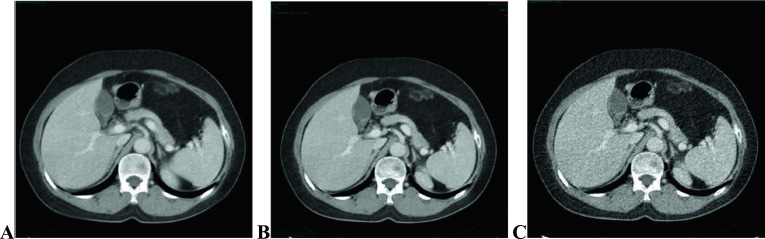
Comparisons of image quality obtained using different reconstruction methods in the portal phase of 70 keV: (**A**) 5-mm ASIR-V40% at 70 keV; (**B**) 1.25-mm ASIR-V40% at 70 keV; (**C**) 1.25-mm DL-H at 70 keV. Both readers agreed that **C** showed a sharper vessel edge, a lower image noise, and a better overall image quality than **A** and **B**, especially **B**

The subjective image quality scores by two senior radiologists are presented in Fig. 2. The kappa test showed good consistencies (kappa > 0.6) between the two readers. According to the 5-point Likert scale, the DLIR-H group was rated the best for overall image quality. The 70-keV 1.25-mm DLIR-H group showed sharper vessel edge, lower image noise, better overall image quality, and the best diagnostic confidence. Pairwise comparisons of subjective scores among the four groups for the same rater were performed and resulted in significant differences (all *P* values < 0.001) (Figs. 3 and 4).

### Diagnostic Performance

#### Lesion Detection

The detection rates for the benign and malignant liver lesions > 1 cm in size were all 100% for the three image groups at 1.25-mm slice thickness (the 70-keV 1.25-mm ASIR-V40% group, the 70-keV 1.25-mm DLIR-M group, and the 70-keV 1.25-mm DLIR-H group). The three groups had a consistent lesion detection rate. For the benign and malignant liver lesions more than 0.5 cm, the lesion detection rates for the three image groups were all 100%. For the lesions smaller than 0.5 cm, the lesion detection rates for the benign and malignant liver lesions were also consistent among the three groups (93.33% for the benign lesions and 92.86% for the malignant lesions) (Table 3).

#### Diagnostic Confidence

For diagnostic confidence, two readers scored 4.64 ± 0.49 and 4.53 ± 0.50 for DLIR-H images, which were higher than those for the other two groups (Table 4). The kappa test showed good consistencies (kappa > 0.6) between the two readers. Both readers confirmed that the DLIR-H group had the highest diagnostic confidence compared with the other two image groups.

## Discussion

DECT is an emerging technology with irreplaceable advantages in reducing x-ray hardening artifacts and CNR, optimizing image quality, and enhancing the display of low vascular lesions; it facilitates disease diagnosis and lesion detection by radiologists [19–21]. It can synthesize monoenergetic images at different energies, which can be used for routine diagnosis such as conventional polyenergetic images acquired at a single x-ray tube potential [22]. Lv et al. found that the images obtained using DECT increased the sensitivity of identifying small hemangiomas and small hepatocellular carcinomas [21].

As demonstrated by Yamada et al. [6, 7], the VMIs at 70 keV had the lowest noise and the highest CNR; they more accurately reflected the true degree of lesion enhancement compared with conventional CT. Recently, the superiority of DLIR has been demonstrated in both single-energy CT and dual-energy CT scans [17, 23, 24]. Although DLIR has been applied to the DECT field, the applications of DLIR with Gemstone Spectral Imaging in the diagnostic performance of the liver are few.

In this study, using ASIR-V40% as the reference, we investigated the potentiality of DLIR in improving image quality and diagnostic performance of hepatic lesions under DECT conditions. The objective and subjective image quality, lesion detection rate, and diagnostic confidence were compared between two reconstruction algorithms (ASIR-V and DLIR) at 70 keV. The image quality involved four image groups: 5-mm 70-keV ASIR-V40% virtual monochromatic spectral (VMS) images, 1.25-mm 70-keV ASIR-V40%, and DLIR-M and DLIR-H VMS images. The diagnostic performance involved the latter three groups. The results showed that DLIR at 70 keV improved image quality and diagnostic performance (diagnostic confidence) of liver lesions compared with ASIR-40% under the same condition. However, in terms of lesion detection rate, it was similar to ASIR-40% with no significant improvement.

The objective assessments of image quality showed that the 1.25-mm 70-keV DLIR-H group had the lowest SD value and the best SNR and CNR compared with ASIR-V40% and DLIR-M under the same condition. No statistically significant difference was observed in SD value, SNR, and CNR compared with the 5-mm 70-keV ASIR-V40% group. The 1.25-mm 70-keV DLIR-H group also had higher subjective image quality scores than the other three groups regarding vessel edge, image noise, overall image quality, and influence on diagnosis. Although the detection rate of liver lesions was consistent among the three groups, both physicians reported that the 1.25-mm 70-keV DLIR-H group had the best diagnostic confidence, which was higher than that in the ASIR-V40% and DLIR-M groups under the same condition.

This study had some limitations. First, the number of liver lesions, especially the malignant lesions, was relatively small. We plan to further expand the sample size to evaluate the liver lesions more systematically and comprehensively in the future. Second, this study analyzed only one VMI at 70 keV and did not compare it with other levels. We will continue to investigate the results of VMIs at different levels combined with DLIR to find the optimal diagnostic VMIs. Third, only standard-dose groups were included in this study; the study lacked a control group in which CT images were achieved under low-dose scanning conditions. We will further explore the ability of DLIR-DECT to reduce the radiation dose.

In conclusion, 70-keV DECT with DLIR-H further reduced image noise and improved image quality compared with 70-keV DECT with ASIR-V40. Furthermore, it improved diagnostic confidence while ensuring a consistent lesion detection rate of liver lesions.

## Abbreviations

CNR: Contrast-to-noise ratio
SNR: Signal-to-noise ratio
VMS: Virtual monochromatic spectral
DECT: Dual-energy computed tomography
DLIR: Deep learning image reconstruction
DLIR-H: Deep learning image reconstruction at high levels
DLIR-M: Deep learning image reconstruction at medium levels
ASIR-V: Adaptive statistical iterative reconstruction-Veo
ROIs: Regions of interest
SD: Standard deviation

## References

[1] Bhandari A, Koppen J, Wastney T, Hacking C (2023). A systematic review and meta-analysis of spectral CT to differentiate focal liver lesions. Clin Radiol.

[2] Marrero JA, Kulik LM, Sirlin CB (2018). Diagnosis, staging, and management of hepatocellular carcinoma: 2018 practice guidance by the American Association for the Study of Liver Diseases. Hepatology.

[3] Li J, Zhao S, Ling Z (2022). Dual-energy computed tomography imaging in early-stage hepatocellular carcinoma: a preliminary study. Contrast Media Mol Imaging.

[4] Coursey CA, Nelson RC, Boll DT (2010). Dual-energy multidetector CT: how does it work, what can it tell us, and when can we use it in abdominopelvic imaging?. Radiographics.

[5] Marin D, Boll DT, Mileto A (2014). State of the art: dual-energy CT of the abdomen. Radiology.

[6] Lv P, Lin XZ, Chen K, Gao J (2012). Spectral CT in patients with small HCC: investigation of image quality and diagnostic accuracy. Eur Radiol.

[7] Yamada Y, Jinzaki M, Tanami Y, Abe T, Kuribayashi S (2012). Virtual monochromatic spectral imaging for the evaluation of hypovascular hepatic metastases: the optimal monochromatic level with fast kilovoltage switching dual-energy computed tomography. Invest Radiol.

[8] Wang JJ, Chi XT, Wang WW, Deng K (2022). Analysis of contrast-enhanced spectral chest CT optimal monochromatic imaging combined with ASIR and ASIR-V. Eur Rev Med Pharmacol Sci.

[9] Yin XP, Zuo ZW, Xu YJ (2017). The optimal monochromatic spectral computed tomographic imaging plus adaptive statistical iterative reconstruction algorithm can improve the superior mesenteric vessel image quality. Eur J Radiol.

[10] Fang T, Deng W, Law MW (2018). Comparison of image quality and radiation exposure between conventional imaging and gemstone spectral imaging in abdominal CT examination. Br J Radiol.

[11] Sato M, Ichikawa Y, Domae K (2022). Deep learning image reconstruction for improving image quality of contrast-enhanced dual-energy CT in abdomen. Eur Radiol.

[12] Kawashima H, Ichikawa K, Takata T (2020). Performance of clinically available deep learning image reconstruction in computed tomography: a phantom study. J Med Imaging (Bellingham).

[13] Lee JE, Choi SY, Hwang JA (2021). The potential for reduced radiation dose from deep learning-based CT image reconstruction: a comparison with filtered back projection and hybrid iterative reconstruction using a phantom. Medicine (Baltimore).

[14] Zhao K, Jiang B, Zhang S (2022). Measurement accuracy and repeatability of RECIST-defined pulmonary lesions and lymph nodes in ultra-low-dose CT based on deep learning image reconstruction. Cancers (Basel).

[15] Wang Y, Zhan H, Hou J (2021). Influence of deep learning image reconstruction and adaptive statistical iterative reconstruction-V on coronary artery calcium quantification. Ann Transl Med.

[16] Benz DC, Benetos G, Rampidis G (2020). Validation of deep-learning image reconstruction for coronary computed tomography angiography: impact on noise, image quality and diagnostic accuracy. J Cardiovasc Comput Tomogr.

[17] Noda Y, Kawai N, Nagata S (2022). Deep learning image reconstruction algorithm for pancreatic protocol dual-energy computed tomography: image quality and quantification of iodine concentration. Eur Radiol.

[18] Agarwal A, Jain M (2008). Multidetector CT portal venography in evaluation of portosystemic collateral vessels. J Med Imaging Radiat Oncol.

[19] Nakamura Y, Higaki T, Honda Y (2021). Advanced CT techniques for assessing hepatocellular carcinoma. Radiol Med.

[20] Hamid S, Nasir MU, So A, Andrews G, Nicolaou S, Qamar SR (2021). Clinical applications of dual-energy CT. Korean J Radiol.

[21] Lv P, Lin XZ, Li J, Li W, Chen K (2011). Differentiation of small hepatic hemangioma from small hepatocellular carcinoma: recently introduced spectral CT method. Radiology.

[22] Mccollough CH, Leng S, Yu L, Fletcher JG (2015). Dual- and multi-energy CT: principles, technical approaches, and clinical applications. Radiology.

[23] Heinrich A, Schenkl S, Buckreus D, Güttler FV, Teichgräber UK (2022). CT-based thermometry with virtual monoenergetic images by dual-energy of fat, muscle and bone using FBP, iterative and deep learning-based reconstruction. Eur Radiol.

[24] Noda Y, Kawai N, Kawamura T (2022). Radiation and iodine dose reduced thoraco-abdomino-pelvic dual-energy CT at 40 keV reconstructed with deep learning image reconstruction. Br J Radiol.

